# Self-association of the Lentivirus protein, Nef

**DOI:** 10.1186/1742-4690-7-77

**Published:** 2010-09-23

**Authors:** Youn Tae Kwak, Alexa Raney, Lillian S Kuo, Sarah J Denial, Brenda RS Temple, J Victor Garcia, John L Foster

**Affiliations:** 1 Division of Infectious Diseases, Center for AIDS Research, University of North Carolina, Chapel Hill, North Carolina 27599-7042, USA; 2 Department of Biochemistry and Biophysics, R. L. Juliano Structural Bioinformatics Core, University of North Carolina, Chapel Hill, North Carolina 27599-7042, USA; 3 Baylor Institute for Immunology Research, 3434 Live Oak, Dallas, TX 75204, USA; 4 Department of Internal Medicine, University of Texas Southwestern Medical Center at Dallas, 5323 Harry Hines Boulevard, Y9.206, Dallas, Texas 75390, USA

## Abstract

**Background:**

The HIV-1 pathogenic factor, Nef, is a multifunctional protein present in the cytosol and on membranes of infected cells. It has been proposed that a spatial and temporal regulation of the conformation of Nef sequentially matches Nef's multiple functions to the process of virion production. Further, it has been suggested that dimerization is required for multiple Nef activities. A dimerization interface has been proposed based on intermolecular contacts between Nefs within hexagonal Nef/FynSH3 crystals. The proposed dimerization interface consists of the hydrophobic B-helix and flanking salt bridges between R105 and D123. Here, we test whether Nef self-association is mediated by this interface and address the overall significance of oligomerization.

**Results:**

By co-immunoprecipitation assays, we demonstrated that HIV-1Nef exists as monomers and oligomers with about half of the Nef protomers oligomerized. Nef oligomers were found to be present in the cytosol and on membranes. Removal of the myristate did not enhance the oligomerization of soluble Nef. Also, SIVNef oligomerizes despite lacking a dimerization interface functionally homologous to that proposed for HIV-1Nef. Moreover, HIV-1Nef and SIVNef form hetero-oligomers demonstrating the existence of homologous oligomerization interfaces that are distinct from that previously proposed (R105-D123). Intracellular cross-linking by formaldehyde confirmed that SF2Nef dimers are present in intact cells, but surprisingly self-association was dependent on R105, but not D123. SIV_MAC239_Nef can be cross-linked at its only cysteine, C55, and SF2Nef is also cross-linked, but at C206 instead of C55, suggesting that Nefs exhibit multiple dimeric structures. ClusPro dimerization analysis of HIV-1Nef homodimers and HIV-1Nef/SIVNef heterodimers identified a new potential dimerization interface, including a dibasic motif at R105-R106 and a six amino acid hydrophobic surface.

**Conclusions:**

We have demonstrated significant levels of intracellular Nef oligomers by immunoprecipitation from cellular extracts. However, our results are contrary to the identification of salt bridges between R105 and D123 as necessary for self-association. Importantly, binding between HIV-1Nef and SIVNef demonstrates evolutionary conservation and therefore significant function(s) for oligomerization. Based on modeling studies of Nef self-association, we propose a new dimerization interface. Finally, our findings support a stochastic model of Nef function with a dispersed intracellular distribution of Nef oligomers.

## Background

The human immunodeficiency virus type I (HIV-1) accessory gene product, Nef, is a myristoylated protein with a decisive role in viral replication and pathogenesis [1-4]. HIV-1Nef has a canonical length of only 206 amino acids but is functionally complex. Simian immunodeficiency virus (SIV) and human immunodeficiency virus type 2 (HIV-2) Nefs are about 50 amino acids longer and are also functionally complex [2]. In both cases functional complexity is reflected in overlapping effector domains that interact with multiple cellular proteins. These interactions bring about abnormal associations of host cell proteins that establish a favorable environment for viral replication [2,5-8]. HIV-1Nef has a structured core (approximately amino acids 62-147 and 179-200), flexible N- and C- termini (2-61, 201-206) and an internal flexible loop (148-178). Homology between HIV-1 and SIV Nefs is largely restricted to the core region [9]. It has been established that Nef can exist as a dimer, and to a much lesser extent a trimer, with the proposed oligomerization domain residing in the core [10-13].

Several groups have investigated the relationships between Nef's cellular localization, oligomerization, and its various activities. Specifically, a monomer of Nef has been proposed to be soluble, compact, and inactive with the myristate, the N-terminal flexible arm (2-62) and the internal flexible loop (148-178) all bound to the Nef core. In this conformation, soluble Nef may be refractory to oligomerization [14,15]. Support for this model comes from the report that in vitro myristoylated full-length Nef is a compact monomer as determined by analytic gel filtration and ultracentrifugation [15]. Insertion of the myristate alkane chain into the membrane would remove it from the Nef core, possibly favoring the dimeric state [15]. Recent studies suggest that an interaction between positively charged residues within 22 amino acids of the N-terminus and the negatively charged surface of intracellular membranes act in concert with the myristoyl group to enhance and stabilize Nef binding to internal membranes [16,17].

The large conformational changes thought to occur upon Nef binding to membrane are the basis of a tripartite regulatory model for Nef linking conformation, cellular localization, and function by Arold and Baur [14]. In this model the initial cytosolic form of Nef is monomeric, non-functional, and described as "closed." Insertion of the myristate group into membrane, and association of the nearby cluster of arginines (R17, R19, R21, and R22) with phospholipids, detaches the flexible N-terminus from the Nef core giving a "semi-open" conformer. Subsequent extension of the flexible internal loop (amino acids 148-178) away from the Nef core gives a fully extended or "open" form of the protein. These changes could uncover Nef dimerization and effector domains in conjunction with the localization of the protein in proximity to its membrane-bound cellular targets [10,17,18]. The model further proposes that the sequence of Nef conformations, closed, semi-open, and open, directs subsets of Nef activities to appear in a temporal progression from synthesis on cytosolic ribosomes to membrane attachment and finally co-internalization with CD4 and other host cell plasma membrane proteins by the endocytotic machinery. In this way, Nef would be able to express functions important for early HIV-1 replication and then shift to functions appropriate for late HIV-1 replication [14]. Arold and Baur rejected an alternate model of Nef function in which discrete conformers of Nef each with a different set of Nef activities occur randomly and simultaneously.

Reports from Arold and co-workers have also suggested an important role for Nef oligomerization in key Nef functions including CD4 downregulation, MHCI downregulation and enhancement of virion infectivity [10,18]. These authors have described an oligomerization surface contained in the second α-helix (αB) of the Nef core (R105-I114) and 9 amino acids in the trailing loop. The basis for this proposed dimeric structure comes from analysis of Nef packing within hexagonal crystals. D123 is considered to be critical for Nef oligomerization since mutating this residue renders the mutant protein defective for all three of the above Nef activities [18]. Poe and Smithgall have recently reported the presence of Nef dimers possibly linked by the 105-123 dimerization interface, but found Nef dimerization to be independent of membrane localization [13,19]. Hence, the studies published to date raise a number of interesting questions regarding the mechanism and possible significance of Nef oligomerization. Crucial to all of these considerations is the robustness of the proposed inhibition of dimerization in the cytosol by the myristate group. If a significant proportion of oligomeric Nef is formed in the cytosol and transiently binds to membrane as a dimer; then a stochastic model is favored over a sequential model.

Here we report that Nef oligomers are present at levels consistent with functional significance. We also report evidence that supports an equilibrium model of different Nef conformations over the previously described sequential regulation model. For the first time, the evolutionally diverged SIVNef and HIVNef have been demonstrated to share at least one oligomerization interface. Finally, our data indicate the presence of one or more Nef oligomeric states in addition to the R105-D123 model previously proposed. Our modeling studies have suggested a new dimerization interface consistent with all of our data.

## Results

### Membrane-bound Nef exists as monomers and dimers

**Figure 1:**
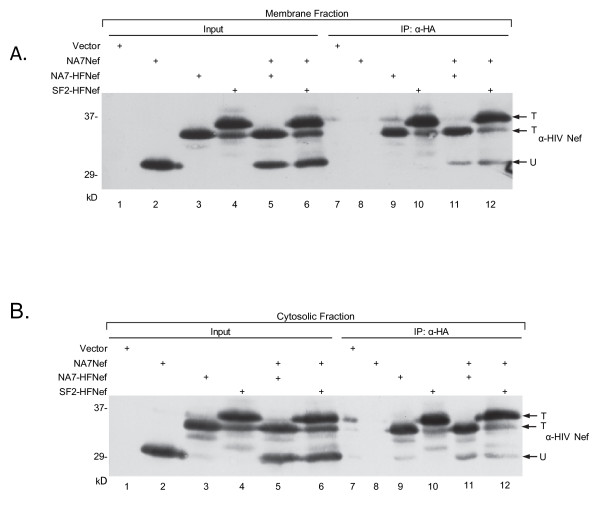
**Assays for Nef oligomers by immunoprecipitation**. **(A) **293T cells were transfected to express untagged NA7Nef (lower molecular weight band, lane 2) and tagged NA7-HFNef and SF2-HFNef (higher molecular weight bands, lanes 3 and 4) singly and in the combinations of a tagged Nef with untagged NA7Nef. The membrane-associated protein fraction was prepared and Nef expression was determined by Western blot analysis (Input, lanes 1-6). Immunoprecipitations were also performed with anti-HA monoclonal antibody and were analyzed by SDS/PAGE and Western blot analysis (IP: α-HA, lanes 7-12). Antibody for the Western blots was sheep anti-HIV-1Nef. *Arrows*, untagged NA7Nef (U) present as hetero-oligomer and total tagged Nef (T). Upper T for SF2-HFNef and lower T for NA7-HFNef. **(B) **Same as in (A) with the corresponding cytoplasmic protein fractions. Note that in (A) the slightly lower molecular weight band present in the SF2-HFNef lanes running the same distance as the NA7-HFNef appears to have been proteolytically modified. In the soluble fractions additional minor proteolytic fragments are present.

**Figure 2:**
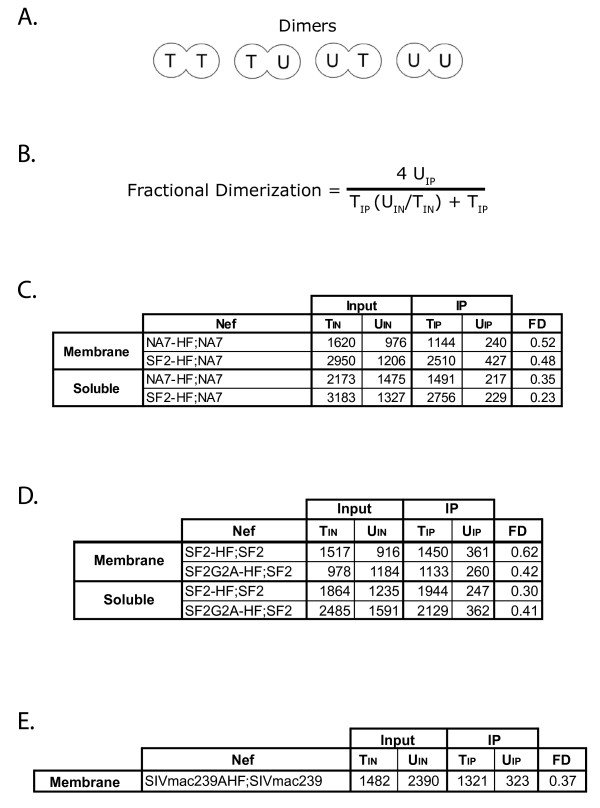
**Estimation of Fractional Dimerization**. **(A) **There are three forms of Nef dimers possible- homodimers of tagged Nefs (T_2_) and untagged (U_2_) Nefs and heterodimers (TU, UT). We have assumed a random assortment of tagged and untagged Nefs with a binomial distribution (1:2:1) of dimeric forms. **(B)** The equation for the fractional dimerization (FD). **(C) **The corrected densities (divided by 1000), determined by ImageJ, for the experiment presented in Figure 1A, B were used to calculate FD's. **(D) **The corrected densities/1000 determined by ImageJ for the experiment presented in Figure 3A, B and the calculated FD's are given. **(E) **The corrected densities/1000 for the experiment in Figure 4A and the FD are presented.

The HIV-1 protein, Nef, is the predominant protein early in HIV-1 replication [20]. Nef has been shown to be distributed between the cytosolic and membrane-bound fractions of the cytoplasm [16,21-25]. The initial questions we addressed were whether or not Nef oligomers are a significant fraction of total membrane-bound Nef and/or soluble Nef. Previous studies have demonstrated the presence of Nef dimers in cells by qualitative assays [12,13]. To determine the extent of Nef dimerization in the soluble and membrane fractions of Nef expressing cells, SF2Nef and NA7Nef were used [26-28]. These well-studied Nefs are highly cross-reactive in our Western blots (Additional File 1; Figure S1). We used NA7-HFNef and SF2-HFNef each with a C-terminal tag containing the HA epitope to be able to resolve these proteins from untagged Nef [29-31]. In Figure 1A we demonstrate that the tagged HIV-1 Nefs, SF2-HFNef and NA7-HFNef, and the untagged NA7Nef are well expressed in membrane fractions of 293T cells (*Input*, lanes 2-4). Note that the tagged proteins are clearly resolvable from untagged NA7Nef by SDS/PAGE. In addition, tagged SF2-HFNef is slightly larger than tagged NA7-HFNef because amino acids 22-25 are repeated in SF2Nef, making it 4 amino acids longer (*Input*, lanes 3 and 4). As expected immunoprecipitation of extract containing untagged NA7Nef with anti-HA monoclonal antibody yielded no detectable untagged Nef (Figure 1A, *IP:α-HA*, lane 8), but NA7-HFNef and SF2-HFNef could be readily pulled down (lanes 9 and 10). Co-expression of NA7-HFNef or SF2-HFNef with untagged NA7Nef allowed us to document the presence of Nef oligomers bound to cellular membranes. Western blots of immunoprecipitated tagged Nefs with anti-Nef antibody revealed the association of NA7Nef with SF2-HFNef and NA7-HFNef (compare lane 9 to 11 and lane 10 to 12, *arrow *labeled "U"). In contrast to the bands for tagged Nefs (T), which represents the total tagged Nef present (monomers and oligomers are indistinguishable), the lower molecular weight band (U) only represents the fraction of untagged NA7Nef that is associated with tagged NA7-HFNef (lane 11) or SF2-HFNef (lane 12). Untagged, homodimeric NA7Nef and monomeric NA7Nef were not immunoprecipitated. Therefore, it is not possible to estimate the fractional dimerization (FD) directly. To estimate FD, we considered Nef to be strictly dimeric since trimers and higher order Nef oligomers are minor species [10-12]. Further, we assumed a binomial distribution between tagged and untagged Nefs (Figure 2A). The fractional dimerization (FD) is ratio of the amount of untagged Nef and tagged Nef present as dimers divided by the total untagged and tagged Nef. Densities were determined and corrected by background subtraction. The corrected densities for the upper band and the lower band in the immunoprecipitated sample lane (T_IP_ and U_IP_, respectively) are used as values that reflect the amount of T and U present in the immunoprecipitate. The entire amount of tagged Nef (T_IP_) in the sample is proportional to the corrected density of the bands marked T (Figure 1, lanes 11 and 12) representing immunoprecipitated tagged Nef. The total amount of untagged Nef cannot be determined directly from U_IP_ (IP: α-HA, lanes 11 and 12). Instead total untagged Nef is determined as the amount of tagged Nef (T_IP_) times the proportion of untagged Nef to tagged Nef. This latter ratio is obtained by dividing the corrected density for U by the corrected density for T from Input (Input, lanes 5 and 6) to give (U_IN_/T_IN_). Total untagged Nef is proportional to T_IP_(U_IN_/T_IN_). Therefore, T_IP_(U_IN_/T_IN_) + T_IP_ is proportional to the total Nef in the sample (denominator in Figure 2B). To obtain an estimate for total dimeric Nef the corrected densities of untagged Nef bands (U_IP_) in the immunoprecipates is the starting point (Figure 1, IP: α-HA, lanes 11 and 12). The dominant species present in this band is the untagged Nef that co-immunoprecipitated with the tagged Nef, but a minor species may be present that is a co-migrating proteolytic fragment from the tagged Nef. This species can be determined from immunoprecipitated Nef that is singly expressed (Figure 1, lanes 9 and 10). The corrected densities of the region of the blot corresponding to the migration of untagged Nef (arrow labeled U) in the lanes only expressing tagged Nef (lanes 9 and 10) were used to correct for the presence of possible proteolytic fragments in co-immunoprecipitating bands. Specifically, to obtain U_IP_ for NA7Nef bound to NA7-HFNef an adjustment for any proteolytic fragment that could be present was obtained by subtracting the corrected density at the same level in lane 9 from "U" in lane 11. Similarly, the U_IP_ for NA7Nef bound to SF2-HFNef was obtained by subtracting the corrected density in lane 10 from "U" in lane 12. These adjusted values are proportional to U_IP_. However, U_IP_ greatly underestimates total dimeric Nef. Clearly, there were equal amounts of tagged and untagged Nef in the immunoprecipitated heterodimers contributing a two-fold correction in the amount of heterodimeric Nef (2XU_IP_). The amount of homodimeric cannot be determined directly. As seen in (A) a random (binomial) distribution of U and T will result in equal or nearly equal amounts of U and T in heterodimers and in homodimers. Varying the ratio of total U, (U_IN_), and total T, (T_IN_), will shift the equilibrium but the fraction of U plus T in heterodimers will remain at approximately 50% of total dimer. In other words, increasing the level of T over U will give higher levels of homodimeric T_2_ and lower levels of homodimeric U_2_ (or if U over T, then U_2_ > T_2_). Therefore, we can conclude that untagged and tagged Nef present in heterodimers (2XU_IP_) is approximately equal to untagged and tagged Nef in homodimers (2XU_IP_). This gives total oligomeric Nef (homodimers and heterodimers): 2XU_IP_ + 2XU_IP_ = 4XU_IP_ (numerator in Figure 2B). The assumption of a binomial distribution is best made under conditions of similar levels of tagged and untagged Nef expression. Experiments in which the U_IN_/T_IN_ ratios exceeded 3-fold were not presented. This co-immunoprecipitation assay gives the first estimates of the level of Nef dimerization in intact cells. However, the complexity of these calculations and the technical difficulties of quantitating immunoprecipitations require cautious interpretation and we do not consider differences in FD of less than two-fold to be compelling. The FDs of membrane-bound NA7-HFNef/NA7Nef (Figure 1A, lane 11) and SF2-HFNef/NA7Nef (Figure 1A, lane 12) were estimated by the formula in Figure 2B to be 0.52, and 0.48, respectively (Figure 2C).

### Nef oligomers are also present in cytosol

The cytosolic fractions corresponding to the membrane-bound fractions presented in Figure 1A were also analyzed (Figure 1B). Unexpectedly, Nef oligomers were present in the cytosol with FD's for NA7-HFNef/NA7Nef and SF2-HF/NA7Nef of 0.35 and 0.23, respectively (Figure 2C). These values suggested a difference between the FDs for soluble Nef versus membrane-bound Nef (Figure 2C). Additional experiments confirmed a difference in FDs for soluble and membrane-bound Nefs (FD_soluble_/FD _membrane _= 0.52 ± 0.05 with n = 4 and p = 0.015 by paired t-test). This 50% lower FD for soluble Nef is qualitatively consistent with the suggestion that the myristate group interacts with the Nef core in soluble Nef and diminishes dimerization [14,15]. However, the fact that this is a partial effect means alternative mechanisms may account for the result.

### The role of the N-terminal myristate in oligomerization

**Figure 3:**
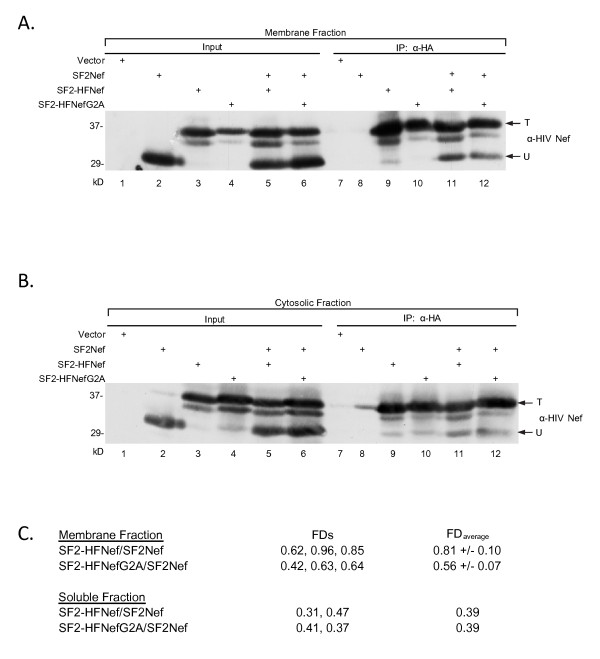
**Myristoylation defective Nef does not exhibit enhanced oligomerization**. **(A, B) **The abilities of untagged SF2Nef to associate with tagged SF2-HFNef or SF2-HFNefG2A in membrane and soluble fractions were compared using the immunoprecipitation assay. The co-immunoprecipitated SF2Nef is indicated by an arrow marked "U" to the right of lane 12 (lanes 11 and 12). The total tagged Nef is indicated by an arrow marked ''T''. (A), Membrane-bound Nef. (B), Cytosolic Nef. **(C) **Data from additional experiments were combined to assess the FDs for the binding of SF2Nef to SF2-HFNef and SF2-HFNefG2A. Analysis was performed as in Figure 2.

Our observation of significant levels of Nef oligomers in the soluble protein fractions suggested that the proposed inhibition of Nef dimerization by its myristate group was weak at best. Nonetheless, if the observed 50% reduction in the fraction of Nef oligomers in the cytosol compared to membrane-bound Nef was the result of inhibition by myristate then the SF2NefG2A mutation should give a higher fraction of Nef dimers in the cytosol than that observed for the parental SF2Nef [14,15]. The capacity of tagged SF2-HFNef and the myristoylation-defective SF2-HFNefG2A to oligomerize with untagged SF2Nef is shown in Figure 3. It has been previously shown that the myristoylation defective Nef associates with cellular membranes, though the majority of this protein is soluble (Additional File 2; Figure S2, *Right *and [16]). We observed in Figure 3A, lane 12 that myristoylation-defective tagged Nef from the membrane fraction associates with untagged SF2Nef, and that this association is similar to that observed with wild type tagged and untagged Nefs (lane 11). For soluble Nef there was also no apparent enhancement of oligomerization as a result of the G2A mutation (Figure 3B, lanes 11 and 12). The FDs for this experiment are given in Figure 2D. The membrane-bound and soluble SF2NefG2A dimerization experiments were repeated and FDs determined (Figure 3C). No difference was observed for FDs of soluble SF2Nef and SF2NefG2A while a small reduction was observed in FDs for membrane-bound SF2NefG2A compared to SF2Nef (FD_G2A_/FD_SF2 _= 0.70 ± 0.05 with n = *3 a*nd p = 0.028 by paired t-test). These combined results suggest that oligomerization is a constitutive property of Nef with little or no impact attributable to the presence of the myristate group. In the absence of compelling evidence that removal of the myristate group on Nef enhances dimerization, a possible explanation for the two fold higher FD for membrane-bound Nef versus soluble Nef could be that the dimeric entity may preferentially bind membrane compared to monomer because it has two membrane association sites.

### SIV_MAC239_Nef forms oligomers

**Figure 4:**
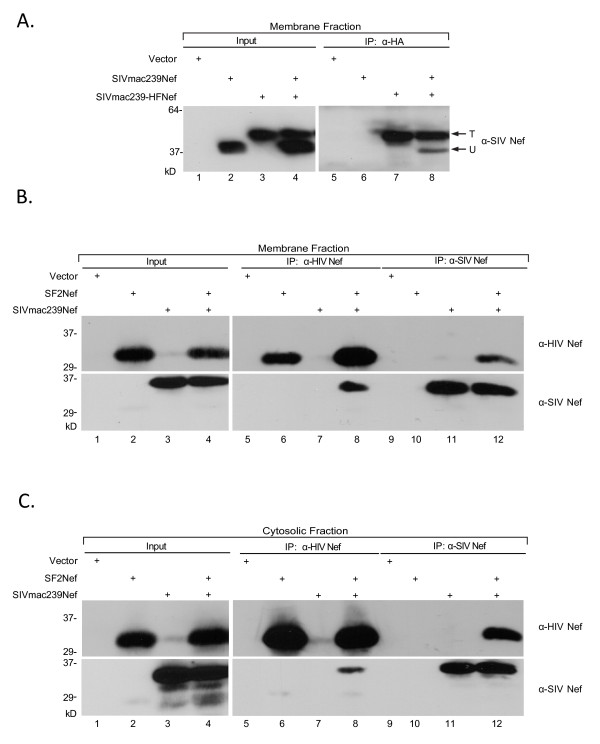
**SIV_MAC239_Nef oligomerizes and also forms heterologous oligomers with SF2Nef**. **(A) **293T cells were transfected to express tagged SIV_MAC239_-HFNef and untagged SIV_MAC239_Nef, singly and in combination. Membrane protein fractions were prepared and Nef expression was determined by Western blot analysis (Input). Immunoprecipitations were performed with anti-HA monoclonal antibody and the immunoprecipitates analyzed by SDS/PAGE followed by Western blotting with monoclonal anti-SIV antibody (IP:α-HA). *Arrows*, total tagged Nef (T) and untagged Nef bound to tagged Nef (U). **(B and C) **293T cells were transfected to express SF2Nef and SIV_MAC239_Nef singly and in combination. Membrane (B) and soluble (C) protein fractions were prepared and Nef expression determined by Western blot analysis with anti-HIVNef monoclonal antibody (Input, upper panels) and anti-SIVNef monoclonal antibody (Input, lower panels). The membrane and soluble fractions were also immunoprecipitated with polyclonal anti-HIV-1Nef (IP: α-HIV-1Nef) and polyclonal anti-SIVNef (IP: α-SIV Nef). The immunoprecipitates were analyzed by Western blot with monoclonal anti-HIV Nef (upper panels) and monoclonal anti-SIVNef (lower panels).

The SIV_MAC239_Nef protein is derived from a proviral molecular clone encoding a virus that is highly pathogenic in rhesus macaques [3]. It has all the major activities of SF2Nef in human cells, despite only 43% identity between the two proteins [2]. Interestingly, Arold et al. have argued that non-conserved residues in SIV and HIV-2 Nefs relative to HIV-1 Nefs (115Y in HIV-1Nef to D or E in SIVNef and 116 H in HIV-1Nef to R in SIVNef) would lead to burying charged residues in the hydrophobic core of the proposed dimerization domain of αB helix [10]. This sequence difference between SIV and HIV-1 Nefs suggested that SIV Nefs would not oligomerize or do so by a different mechanism to HIV-1Nefs. In an analogous experiment to Figure 1 we asked the question if tagged SIV_MAC239_-HFNef associates with untagged SIV_MAC239_Nef (Figure 4A). We prepared membrane fractions and analyzed Nef expression by Western blot analysis. We also performed immunoprecipitations with anti-HA antibody. SIV_MAC239_Nef can be resolved from SIV_MAC239_-HFNef by SDS/PAGE (Figure 4A, *Input*, lanes 2-4) and anti-HA antibody failed to immunoprecipitate SIV_MAC239_Nef (Figure 4A, *IP: α-HA*, lane 6). However, when SIV_MAC239_Nef is co-expressed with SIV_MAC239_-HFNef, followed by immunoprecipitation with anti-HA antibody, untagged SIV_MAC239_Nef is brought down in association with tagged SIV_MAC239_-HFNef (lane 8, *lower arrow*). The relative intensities of the T and U bands correspond to FD = 0.37 (Figure 2E). This striking observation demonstrated the existence of SIV_MAC239_Nef oligomerization that is unrelated to the previously proposed dimerization interface [10,18]. If SIVNef oligomerization is by a different mechanism to HIV-1Nef then one would not expect the two proteins to associate. In a preliminary experiment we observed that following co-expression SIV_MAC239_-HFNef did indeed pull down SF2Nef which is an HIV-1Nef (not shown). Accordingly, we have investigated the heterologous association of SF2Nef and SIV_MAC239_Nef without tags that were specifically immunoprecipitated with non-crossreacting antibodies. SF2Nef and SIV_MAC239_Nef were expressed either singly or together. Membrane and cytosolic protein fractions were prepared and analyzed in Figure 4B and 4C, respectively. The levels of the two Nef proteins are shown in Figure 4B and 4C*Input*. The analyses of the two immunoprecipitations, with either anti-HIVNef or anti-SIV Nef antisera, are presented in Figure 4B and 4C (*IP: α-HIV Nef *and *IP: α-SIV Nef*). The anti-HIVNef antiserum did not precipitate SIV_MAC239_Nef (Figures 4B and 4C, lane 7, lower panels) and the anti-SIVNef antiserum did not precipitate SF2Nef (Figures 4B and 4C, lane 10, upper panels). Immunoprecipitations of extracts containing the co-expressed Nefs brought down the non-reactive protein, demonstrating an association between HIVNef and SIVNef (Figure 4B and 4C, *IP: α-HIV Nef*, compare in the lower panels, lanes 7 and 8; and *IP: α-SIV Nef*, compare in the upper panels, lanes 10 and 12). The observed heterologous association between SF2Nef and SIV_MAC239_Nef implies there are homologous dimerization interfaces present in these Nefs that are distinct from the previously proposed dimerization interface (R105-D123).

### Nef dimers in intact cells

**Figure 5:**
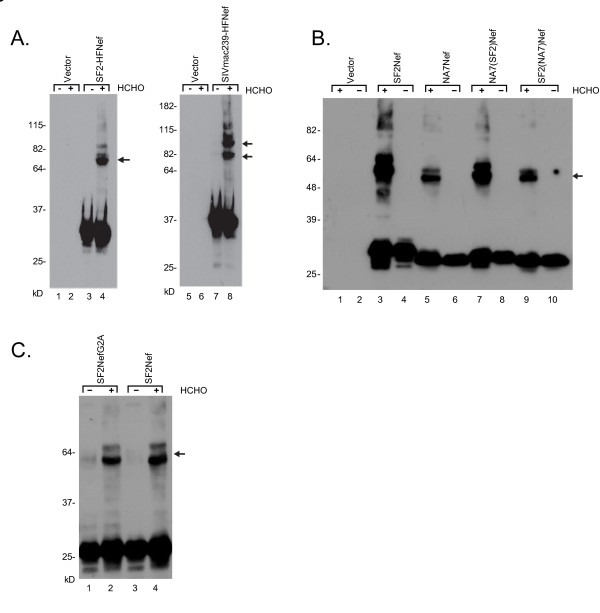
**Cross-linking Nef dimers in intact cells with formaldehyde**. **(A) **293T cells were transfected to express SF2-HFNef or SIV_MAC239_-HFNef. Intact cells were incubated with 0.5% formaldehyde for 10 minutes at room temperature. Clarified whole cell lysates were prepared and Nef detected by Western blot analysis. (-), incubation without formaldehyde. (+), incubation with formaldehyde. *Arrows*, cross-linked Nef dimers. **(B) **Assay of intracellular oligomerization with NA7Nef/SF2Nef chimeras. The chimeric expression vectors were made by substituting coding sequence for amino acids 24-200 SF2Nef into NA7Nef and the same coding sequence for NA7Nef into SF2Nef. Oligomerization was assayed by formaldehyde cross-linking in intact cells as in (A). NA7(SF2)Nef, NA7Nef with coding sequence for amino acids 24-200 replaced by coding sequence for the same amino acids from SF2Nef. SF2(NA7)Nef, the reciprocal construct as NA7(SF2)Nef. (-), incubation without formaldehyde; (+), incubation with formaldehyde. *Arrow*, cross-linked Nef dimer. **(C)**, The ability of SF2NefG2A to associate with itself was compared to SF2Nef by the formaldehyde cross-linking assay with intact cells. Arrow to the right of lane 4 indicates cross-linked Nef (lanes 2 and 4). (+), Cells incubated with 0.5% formaldehyde for 10 minutes at room temperature. (-), Cells incubated in the absence of formaldehyde.

We next considered the question of Nef self-association in intact cells. Formaldehyde readily penetrates the cell surface membrane and cross-links intracellular proteins at lysines [32]. First, we optimized the detection of Nef oligomers in intact cells by treating SF2Nef-expressing 293T cells with increasing levels of formaldehyde followed by analysis of cell-free lysates by SDS/PAGE and Western blot. The optimal concentration of formaldehyde for cross-linking Nef dimers was determined to be 0.5% (not shown). In Figure 5A we demonstrate that SF2Nef can be cross-linked to yield a single major species under these conditions. Our results are similar to the findings of Kienzle et al. with HIV-1_LAI _Nef cross-linked with bis(sulfosuccinimidyl)suberate in intact insect cells [12]. Interestingly, under the same experimental conditions SIV_MAC239_Nef produced two major cross-linked forms. Whether both of these forms represent cross-linked SIV_MAC239_Nef dimers at different lysine residues or whether one is dimeric Nef and the other the result of cross-linking to a cellular protein is not known (See **Discussion**).

The SF2Nef dimers that we observed by intracellular cross-linking with formaldehyde are present at 15% or less of the total Nef. The likely explanation is that much of the intracellular dimer is either not cross-linked or non-specifically cross-linked. Concentrations of formaldehyde higher than 0.5% resulted in weaker dimer bands due to non-specific cross-linking (not shown). Therefore, it is clear that the results of the formaldehyde cross-linking assay are not quantitative and may not be comparable to the immunoprecipitation assay. To test if the two assays yield similar results we took advantage of a tendency we noted in the immunoprecipitation assay for 2-fold higher FD's for oligomers composed of SF2Nef and SF2-HFNef relative to complexes containing NA7Nef or NA7-HFNef with each other or in hetero-dimeric combinations with SF2-HFNef or SF2Nef, respectively. To carefully compare the strength of self-interactions between SF2Nef and NA7Nef, we determined the FD's for the four possible combinations of SF2-HFNef with SF2Nef or NA7Nef, and NA7-HFNef with SF2Nef or NA7Nef by the immunoprecipitation assay of membrane-bound Nef (not shown). These FDs confirmed the initial observation. Combining all our relevant data, we found the FD for SF2Nef homodimers to be 0.71 ± 0.10 (n = 5) and for SF2Nef/NA7Nef heterodimers plus NA7Nef homodimers to be 0.30 ± 0.09 (n = 5). This difference was statistically significant (p = 0.032 by Mann Whitney test), and suggested that NA7Nef lacks one or more structural features present in the SF2Nef that stabilize dimerization. The observed difference in FDs allowed us to address whether the formaldehyde cross-linking assay was detecting the same oligomers as the immunoprecipitation assay. In that case there should also be a reduction in NA7Nef complexes relative to SF2Nef by formaldehyde cross-linking. The results in Figure 5B, lanes 3 and 5, demonstrate that this is in fact the case. Taking advantage of our finding we swapped the core domains of SF2Nef and NA7Nef to determine if the core sequences accounted for the observed differences in self-association. In Figure 5B, lanes 7 and 9 we observed that the NA7(SF2)Nef chimera with the SF2Nef core exhibited greater cross-linking than the SF2(NA7)Nef chimera with the NA7Nef core. These results strongly suggest that the formaldehyde cross-linking assay and the immunoprecipitation assay are detecting the same Nef oligomers and that oligomerization is dependent on core sequences.

The important finding that the immunoprecipitation assay and the intracellular cross-linking assay give similar results for SF2Nef and NA7Nef self-association led us to determine if the results of Figure 3 would also be confirmed. In Figure 5C we observed that SF2NefG2A did not exhibit enhanced dimerization by the formaldehyde cross-linking assay consistent with the results of the immunoprecipitation assay.

### Can Nef dimers be cross-linked at cysteines?

**Figure 6:**
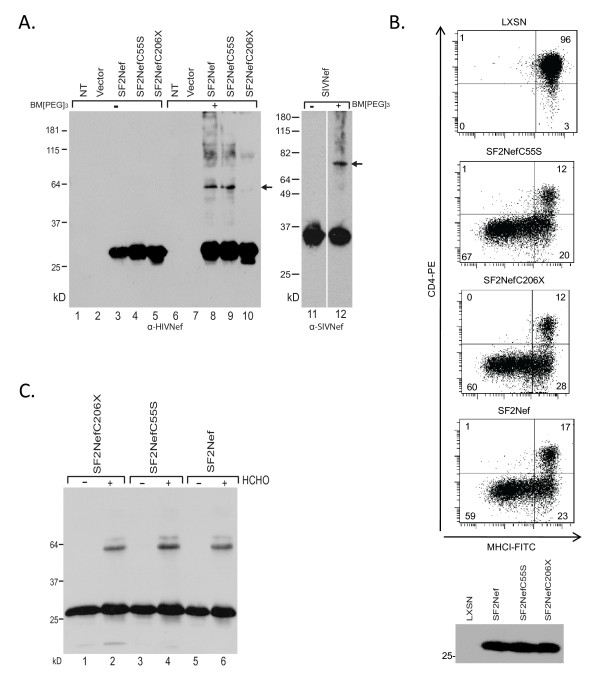
**Cross-linking Nefs dimers with BM[PEG]_3_**. **(A) **293T cells were transfected to express SF2Nef, SF2NefC55S and SF2NefC206X (*left*, lanes 1-10), and SIV_MAC239_Nef (*right*, lanes 11 and 12). Clarified whole cell Iysates were incubated with BM[PEG]_3_. Nef was then detected by Western blot analysis. (-), clarified whole cell Iysates incubated in the absence of BM[PEG]_3 _for 1 hour at room temperature (lanes 1-5 and 11). (+), clarified whole cell Iysates incubated in the presence of 0.5 mM BM[PEG]_3 _for 1 hour at room temperature (lanes 6-10 and 12). The presence of crosslinked Nefs was detected by Western Blot analysis. NT, 293T cells not transfected. **(B) **SF2Nef, SF2C55S, and SF2C206X were assayed for CD4 downregulation and MHCI downregulation in transduced CEM cells (*top*). The level of expression of SF2Nef, SF2NefC55S and SF2NefC206X in CEM cells was determined by Western blot analysis (*bottom*). **(C) **Lysates from transfected 293T cells expressing SF2NefC206X, SF2NefC55S, and SF2Nef were cross-linked by 0.5% formaldehyde.

It has been reported that air oxidation of HIV-2_NIH-Z _Nef and HIV-1_LAI _Nef results in the formation of cross-linked dimers [11,12]. We have confirmed these observations with SIV_MAC239_Nef and SF2Nef (Additional File 3; Figure S3). The observed dimers were formed by intermolecular cystine bonds, as demonstrated by their elimination in the presence of high concentrations of 2-mercaptoethanol (Additional File 3; Figure S3, lanes 1, 2, and 9). The S-S bond formation in SIV_MAC239_Nef dimers must occur at cysteine 55, since it is the only cysteine. However, both HIV-1_LAI_Nef and SF2Nef have cysteines at 55, 142, and 206- the C-terminal amino acid. To determine which of these cysteines are involved in cross-linking, we mutated each of the cysteines, SF2NefC55S, SF2NefC142S, and SF2Nef with C206 deleted (SF2NefC206X). Clarified cell-free extracts containing these Nefs were cross-linked with BM[PEG]_3_. In Figure 6A, *left*, SF2Nef and SF2NefC55S exhibited cross-linking, but SF2NefC206X did not. BM[PEG]_3 _cross-linked SIV_MAC239_Nef at C55 (Figure 6A, *right*). The divergent result that SIV_MAC239_Nef is cross-linked at C55, but SF2Nef is not, suggests a dimeric interface present in SIV_MAC239_Nef that is absent in SF2Nef. This dimeric interface unique to SIV_MAC239_Nef would be separate from the dimerization interface responsible for heterologous association.

In Figure 6B, *top*, we checked the impact of C55S and C206X on the ability of SF2Nef to downregulate CD4 downregulation and MHCI downregulation; both mutants were found to be functional. Neither mutation reduced the level of Nef expression relative to SF2Nef (Figure 6B, *bottom*). In contrast, SF2NefC142S was significantly less stable than the other two cysteine mutants (not shown), confirming previous reports [33,34]. This mutant was partially defective for MHCI downregulation but fully functional for CD4 downregulation. Since MHCI downregulation is more sensitive to reduced levels of Nef expression than CD4 downregulation, this result is expected [31]. Residue 142 is completely buried in the hydrophobic core of Nef and unlikely to be involved in intermolecular S-S bonds between native Nefs [14].

On the basis of these cross-linking experiments, it appears that SF2Nef exhibits a dimeric structure that brings C-terminal regions into proximity. Inspection of space filling models of Nef dimers associated at the previously proposed R105-D123 interface shows the two C-terminal cysteines to be distant from each other (not shown). A second Nef/Nef interaction surface found in cubic crystals of Nef extends from R188 to E197 plus F139 but does not include C206 [10]. This surface is viewed by Arold et al. as a biologically irrelevant result of crystal packing [10]. However, it is clear from inspection of Nef crystal structures that if Nef self-association was mediated by this interface in the C-terminal domain, then cross-linking of C206 by the BM[PEG]_3 _reagent would be possible (not shown). These considerations suggest that the cysteine cross-linking result in Figure 6A may reflect proximity of C-terminal cysteines in the SF2Nef BM[PEG]_3 _cross-linked dimer, but not a structural role in dimerization per se. To test this conclusion we cross-linked SF2Nef, SF2NefC55S, and SF2NefC206X with formaldehyde and found no impact of cysteine mutations on dimerization (Figure 6C). Therefore, we interpret our cysteine cross-linking results as reflecting the location of an interaction interface but not defining C206 as a critical structural element for dimerization. It is interesting to note in this regard that 15% of subtype B Nefs have no C-terminal cysteine but a termination codon instead (TGY to TGA).

### Salt bridges flanking the αB helix are not necessary for oligomerization

It has been proposed that R105 forms salt bridges with D123 on the flanks of the amphipathic αB helix, and that mutation of D123 disrupts oligomerization [10,18]. Several groups have reported multiple functional consequences of mutating D123, including CD4 downregulation, MHCI downregulation, and enhancement of virion infectivity. The multiple defective phenotype of D123 mutants has been attributed to disruption of Nef dimerization [18]. The conservative mutation D123E gives the same defective phenotype as non-conservative mutations, and has also been found to enhance p21-activated protein kinase 2 (PAK2) activation relative to unmodified SF2Nef [8]. The significant impacts on four separate Nef activities define a distinctly complex D123 mutant phenotype [8,14,18,28,35,36]. However, the fact that aspartate replaced by the isoelectric glutamate generates the equivalent phenotype as non-conservative mutations suggests that the isoelectric mutation involves disruption of precise structural considerations beyond mere charge interactions. A phenotypic impact as a result of mutating D123 to glutamate is consistent with its extreme conservation (> 99%). In contrast, HIV-1 subtype B Nefs are weakly conserved at position 105 with 71% lysine, 22% arginine, and 6% glutamine [8].

**Figure 7:**
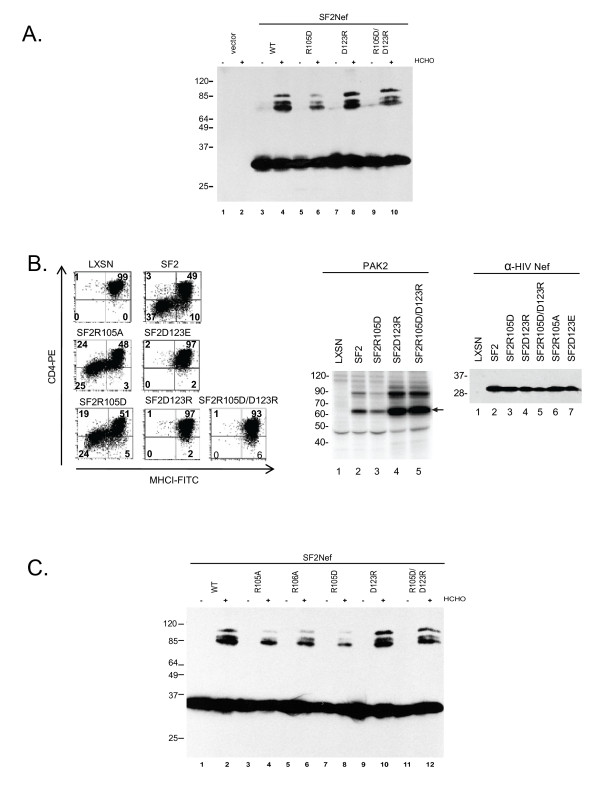
**R105 and R106 are critical for Nef oligomerization**. **(A)**, 293T cells were transfected to express SF2-HFNef (WT), SF2-HFNefR105D (R105D), SF2-HFNefD123R (D123R), and SF2-HFNefR105D/D123R (R105D/D123R). The presence of Nef dimers was detected by treatment of intact cells with 0.5% formaldehyde (+). No formaldehyde (-). **(B) **Transduced CEM cells expressing SF2Nef, SF2NefR105A, SF2NefD123E, SF2NefR105D, SF2NefD123R, or SF2NefR105D/D123R plus the vector (LXSN) positive control were assayed for CD4 and MHCI cell surface expression by flow cytometry analysis *(left) *, PAK2 activation *(middle)*, and level of Nef expression by Western blot analysis *(right)*. **(C) **Role of R105 and R106 in dimerization was investigated as in (A). SF2-HFNefR105A (R105A), SF2-HFNefR106A (R106A).

The impact of mutating R105 and D123 on the level of Nef dimers in intact cells is presented in Figure 7A. We initially used the replacement mutations R105D, D123R, and R105D/D123R. We observed that SF2NefR105D is defective in dimerization consistent with the salt bridge model but SF2NefD123R gave no effect on dimerization (Figure 7A). The double mutation was nearly wild type for dimerization. To assess this unexpected result that R105D was defective for dimerization but D123R was not, we investigated the functional impacts of these mutations (Figure 7B, *left *and *middle*). SF2NefD123R is fully defective for CD4 downregulation and MHCI downregulation but enhanced for PAK2 activation as previously reported for SF2NefD123E [8]. Thus, the drastic mutation of D123R gave the equivalent phenotype as the conservative mutation D123E [8]. The partially functional defects previously reported for R105A were also observed for the charge reversal mutation R105D (Figure 7B, *left, middle*, and [8]). The double mutation SF2NefR105D/D123R that could in theory interact with itself failed to restore Nef function in these assays (Figure 7B, *left *and *middle*). Instead the double mutant gave the complete D123 mutant phenotype of no CD4 or MHCI downregulation, but strong activation of PAK2. None of the mutations resulted in an unstable protein (Figure 7B, *right*). These observations clearly dissociate the D123 and R105 mutant phenotypes. The combined import of the present investigation with the previously published functional results of O'Neill, et al. [8] demonstrates that loss of a salt bridge does not account for the distinct, multi-functional defects that result from mutating D123 or R105.

The replacement mutations for R105 and D123 were designed to test the proposal that R105 and D123 were components of a critical salt bridge within a functionally significant dimerization domain. Since our results failed to verify the salt bridge model, we considered alternative explanations for the roles of R105 and D123. One explanation is that mutation of D123 locks Nef into a conformation that is competent for dimerization, but nonetheless different from the conformation of the bulk of wild type SF2Nef. This hypothesis is based on the fact that adding the R105D mutation to give the double mutant has little effect on dimerization per se, and no effect on the functional properties of Nef mutated at D123.

We next considered a role for R105 in dimerization of wild type Nef distinct from D123. Also, of potential interest was the adjacent R106. Unlike R105 R106 is highly conserved (99%). The isoelectric mutation of R106 to lysine, in analogy to D123E, gives a complex phenotype that is quite different from D123E. As previously reported, R106K is highly defective for MHCI downregulation, partially defective for PAK2 activation but functional for CD4 downregulation and enhancement of infectivity. R106A has a more drastic phenotype- defective for MHCI downregulation, fully defective for PAK2 activation, partially defective for CD4 downregulation, and fully defective for enhancement of infectivity. R106L like R106A is fully defective for MHCI downregulation and PAK2 activation but also fully defective for CD4 downregulation [8]. To try and discern if dimerization possibly plays a role in these complex phenotypes we compared the effects of mutating R105 and R106 (R106A, R105A, and R105D) to mutating D123 (D123R and R105D/D123R). In Figure 7C the R105A and the R106A mutant proteins had reduced levels of Nef dimerization, though not to the extent of the reduction observed for R105D. This suggests that the region of Nef containing R105 and R106 is significant for dimerization and that having the negatively charged D at position 105 may disrupt the normal interactions of the positively charged R106. It is important to note that 93% of Nefs are lysine or arginine at residue 105 and 99% arginine at 106 suggesting that a +2 charge for these two adjacent residues is necessary for optimal function.

### Modeling Nef dimerization

To gain further insight into potential Nef dimerization interfaces, we employed ClusPro 2.0 http://cluspro.bu.edu for a rigid body docking analysis of Nef. The structure of the monomer used in the ClusPro search, was extracted from the hexagonal crystal structure of Nef (PDB ID 1AVZ, Chain A). For our initial ClusPro analysis, we used the same structure of the Nef monomer as both receptor and ligand. The Nef-Nef interface in the crystal structure providing our search model is the basis for the proposal that the R105-D123 interaction forms a critical component of the dimerization interface. Thus, the Nef structure employed in the ClusPro analysis has R105 and D123 side chains in positions appropriate and optimal for formation of the proposed dimerization interface involving the R105-D123 salt bridge. We chose the 10 most strongly interacting models of dimeric Nef structures for analysis. Seven of the structures involved at least part of the αB helix hydrophobic domain. Interestingly, none of the 10 models included the R105/D123 salt bridge that was evident in the crystal structure.

**Figure 8:**
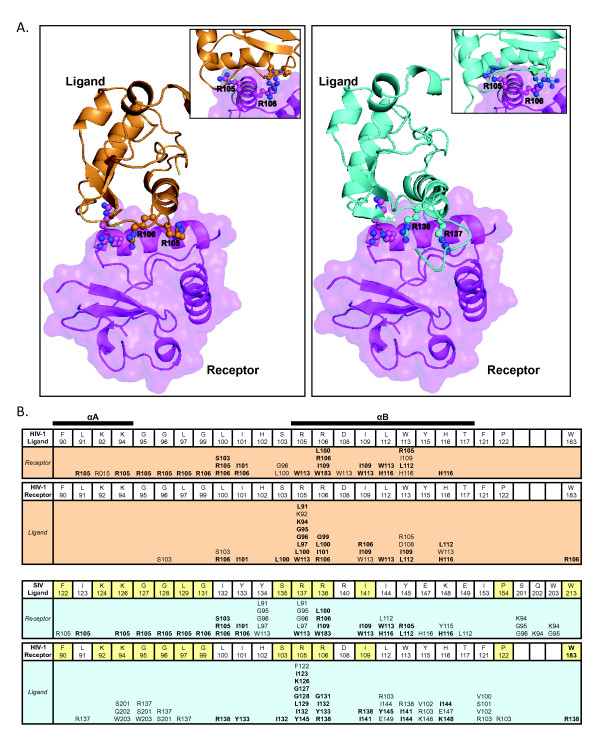
**Three dimensional models of HIV-1 Nef homodimer and HIV-1 Nef/SIV_MAC239_Nef heterodimer**. **(A) **Three dimensional representation of HIV-1Nef interacting with itself (*left*) and SIV_MAC239_Nef *(right)*, identified by rigid body docking using ClusPro. In both docking experiments, HIV-1Nef (PDB 1AVZ, Chain A) was entered as receptor (*left *and *right*- magenta cartoon and transparent surface). In the homodimerization docking, the same Nef was entered as ligand (*left*, *light orange *cartoon). In the heterodimerization docking SIV_MAC239_Nef (PDB 3IK5, Chain A) was entered as ligand (*right*, *cyan *cartoon). The models shown here represent the two complexes generated by ClusPro in which SIV_MAC239_Nef and HIV-1Nef interact with the receptor HIV-1Nef in a nearly identical manner. R105 and R106 for HIV-1Nef and SIV_MAC239_Nef R137 and R138 are represented as ball and stick. Inserts show receptor R105 inserted into a pocket of ligand Nef. **(B) **Residues interacting in the interface of the HIV-1Nef homodimer, and residues interacting in the interface of the HIV-1Nef/SIV_MAC239_Nef heterodimer presented in (A). The three rows of amino acids from F90 to W183 for HIV-1Nef and F122 to W213 for SIV_MAC239_Nef contain all of the residues present at the two dimer interfaces. In the case of SIV_MAC239_Nef, the residues are orthologs of HIV-1Nef residues. In the heterodimer section (*lower half*), the HIV-1Nef and SIV_MAC239_Nef residues that are identical between the two proteins are shaded yellow. Under each boxed residue are the residue or residues that interact with the designated amino acid. Identical interactions between the homodimer and the heterodimer are in bold font. Amino acids S201, Q202, and W203 in SIV_MAC239_Nef correspond to HIV-1Nef internal loop amino acids 171-173 which are deleted in the HIV-1Nef core construct. The extents of conservation at each amino acid position presented in (B) for HIV-1 subtype B Nefs are: F90, 99%; L91, 98%; K92, 98% for R or K; K94, 95%; G95, 99%; G96, 99%; L97, 99%; G99, 99%; L100, 99% for L, I, or M; I101, 98% for I or V; S103, 98%; R105, 99% for K, R, or Q; R106, 99%; D108, 99% for D or E; I109, 99%; L112, 99%; W113, 99%; Y115, 97%; H116, 99% for H or N; T117, 99%; F121, 99%; P122, 99%; W183, 99%. All positions with multiple residues only have amino acids that have positive BLOSUM62 scores (*53*).

We developed three criteria to select the model most likely to be of biological significance from among the seven potential models. First, a model must involve R105 and R106, but exclude D123. The second criterion was that that the model should involve highly conserved residues within Nef. The third criterion required that the potential interface found in the HIV-1Nef homodimer ClusPro analysis also be found in a second ClusPro analysis of the HIV-1Nef and SIV_MAC239_Nef heterodimer. This third criterion became possible with the publication of the structure of the SIV_MAC239_Nef core [37]. We have shown here that SF2Nef associates with both SIV_MAC239_Nef and itself, presumably using homologous interfaces. This implies that there should be a similar model for both the homo- and heterodimer. In the ClusPro analysis to search for heterodimer interfaces, SIV_MAC239_Nef was the ligand and HIV-1Nef was the receptor. As with the HIV-1Nef/HIV-1Nef analysis, the ten most strongly interacting models were inspected. Comparisons between the homodimer and the heterodimer models gave a pair which fit the above three critera (Figure 8A). The positions of the ligand HIV-1Nef, (Figure 8A; cartoon- *left*, *light orange*) and ligand SIV_MAC239_Nef (Figure 8A; cartoon: *right*, *cyan*) relative to the receptor HIV-1Nef (cartoon plus transparent surface: *magenta*) are strikingly similar. None of the other homodimer models matched any of the heterodimer models to give a strikingly matched pair.

For the Nef homodimer (Figure 8B, *light orange *background) the residues that interact between the Nef monomers are presented. For example, ligand R106 interacts with L100, R106, I109, and W183 in β5 (8B; *light orange *background). In total, the ligand HIV-1Nef has 17 residues that interact with the receptor Nef, 14 of which react with the same side chains in receptor Nef as residues in SIV_MAC239_Nef (identical interactions shown in bold, Figure 8B). In the homodimer receptor, 11 residues interact with the ligand with ten of these residues matching ligand residues in SIV_MAC239_Nef residues.

In the HIV-1Nef/SIV_MAC239_Nef heterodimer (Figure 8B, *light blue *background) the ligand SIV_MAC239_Nef has 21 residues that interact with the receptor Nef compared to 17 for the HIV-1Nef ligand. Three of the four extra residues (S201, Q202, and W203) are from the C-terminal portion of the internal loop that is present in SIV_MAC239_Nef core construct that is deleted in the HIV-1 Nef core. In the heterodimer receptor, 18 residues interact with the ligand SIV_MAC239_Nef compared to 11 for the HIV-1Nef homodimer receptor. The seven extra amino acids mostly interact with residues in the SIV_MAC239_Nef ligand that are not present in the Nef core structure, accounting for 12 of 16 interactions. The extra SIV_MAC239_Nef residues are in internal loop or the N-terminal side of PQVPLR near the beginning of the Nef core region. Potentially, the agreement between heterodimer and homodimer would be even greater if the Nef structure contained the extra residues.

**Figure 9:**
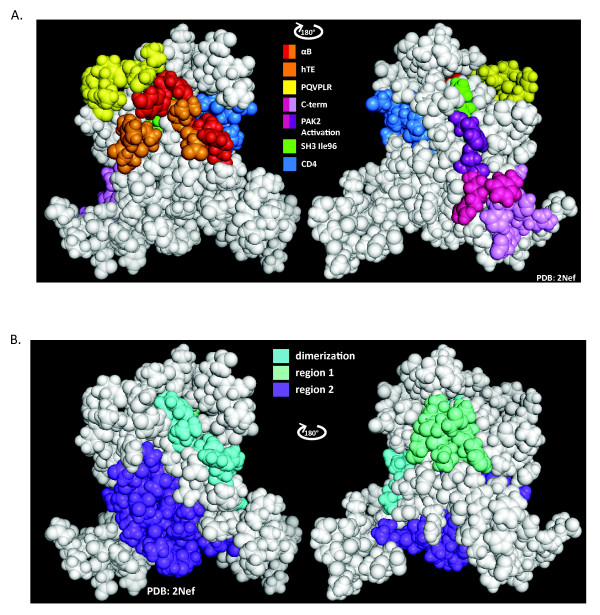
**Three dimensional representations of the functional regions of Nef that interact with host cell proteins or represent putative dimerization domains**. **(A) *left***, *Red *and *Orange*, αB and flanking loop amino acids of the Nef/Nef interface in hexagonal crystals of PDB 1AVZ (amino acids D108, L112, Y115, H116, F121, P122, D123); *Orange*, residues that interact with human thioesterase (amino acids D108, L112, F121, P122, D123); *Yellow*, PQVPLR in the proline helix of the Nef SH3 binding domain (amino acids 72-77); *Blue*, residues that interact with the cytoplasmic tail of CD4 (amino acids W57, L58, E59, G95, L97, R106, L110); ***right***, Image is rotated 180 degrees. *Lilac *and *Magenta*, C-terminal Nef/Nef interface seen in cubic crystals of PDB 1AVV (amino acids F139, R188, F191, H192, H193, R196, E197); *Magenta and Purple*, residues known to play a role in PAK-2 activation (amino acids H89, S187, R188, F191); *Green*, residues that form a hydrophobic pocket interacting with Ile96 of the RT loop of the Hck SH3 domain (amino acids L87, F90, W113, I114). **(B) *left***, *Cyan*, critical residues identified in the protein-protein docking experiments, showcased in Figure 8. Only residues that interact in both the receptor and ligand in both the homodimeric and heterodimeric models are indicated (amino acids L100, I101, R105, R106, I109, L112, W113, H116); *blue violet*, one of two regions (region 2) of HIV-1Nef that is highly conserved in SIV Nef with 22 out of 27 identities (amino acids 122 to 148). **right**, *Seafoam green*, second of two regions (region 1) of HIV-1 Nef that is highly conserved in SIV Nef with 11 out of 12 identities (amino acids 88-99).

An interesting feature of both models is the insertion of R105 of the receptor into a pocket in the ligand formed by C-terminal end of the αA helix and the trailing loop (Figure 8A, insets). The importance of R105 is emphasized by the fact that R105 in both receptors and R105/R137 in the ligands contribute to the interface (Figure 8B). There are 7 additional residues that are also part of the dimer interface in all four Nefs in the two models. In HIV-Nef these are located just prior to αB (L100, I101), are contained in αB (R105, R106, I109, L112, W113), or just past the C-terminus of αB (H116). These eight residues overlap with previously proposed Nef interaction interfaces [10,35]. These are the R105-D123 crystal interface (Figure 9A, *left*- *Red *and *Orange*), and the thioesterase II interacting region which is contained within R105-D123 (Figure 9A, *left*, *Orange*). The SIVNef/HIV-1Nef binding interface proposed in this report is indicated in Figure 9B, *left*, *Cyan*. Comparing Figures 9A, *left *and 9B, *left *demonstrates the substantial overlap between the proposed SIVNef/HIVNef interface and the previously proposed interfaces. In Figure 9B, *right*, one of the highly conserved regions (region 1) between SIVNef and HIV-1Nef (*Seafoam Green*) is indicated. This region is adjacent to our proposed SIVNef/HIV-1Nef interaction surface and interacts extensively with R105 and R106 of the ligand in the two ClusPro models. Additional reported interfaces are shown in Figure 9A, *right *[8,10,38,39]. Not shown are additional interactions of Nef's flexible internal loop with AP-1 and AP-2 and the N-terminal arm with cellular membranes and the cytoplasmic tail of MHCI [6,7,16,17,40,41]. The close packing of overlapping domains with the potential for protein/protein interactions creates a remarkable structure/function complexity. Ternary complexes and allostery have recently been proposed for Nef and may be common aspects of this protein's ability to enhance viral fitness [40-42]. Our results demonstrate that oligomerization is a third significant mechanism for achieving unprecedented structure/function complexity.

## Discussion

Prior to the present report, the extent of Nef oligomerization was not known nor had the regulatory model of Arold and Baur been directly tested [14]. Poe and Smithgall reported the presence of Nef dimers in intact cells by bimolecular fluoresecence complementation but the fractional dimerization was not assessed [13]. To investigate Nef self-association, we have employed two assays. First, the immunoprecipitation assay which utilizes a tagged Nef and an untagged Nef. The tag allowed us to differentiate between the two otherwise identical Nefs immunologically and by different mobilities on SDS/PAGE electrophoresis. This approach allowed us to demonstrate, for the first time, that Nef oligomers are distributed between the membrane and cytosol compartments of the cell. We placed the tag at the C-terminus to preserve Nef myristoylation. The results from this assay were compared to intracellular cross-linking by formaldehyde. We have also demonstrated for the first time that SF2Nef can be cross-linked by BM[PEG]_3_. This cysteine reactive agent cross-linked only at C206 and provided new information of a structural nature regarding the location of a Nef dimerization interface. With these new approaches we have investigated previously unaddressed questions regarding Nef oligomerization, and identified R105 and R106 as important constituents of a dimeric interface.

### What is the proportion of Nef that is oligomeric in cells?

With the immunoprecipitation assay we found approximately two thirds of membrane-bound SF2Nef is oligomerized. Lower levels of oligomerization by half were observed for membrane-bound NA7Nef and SIV_MAC239_Nef. All three of these Nefs are highly functional, well-studied alleles [2,26-28,43] though NA7Nef exhibits weak PAK2 activation relative to SF2Nef [31]. In the case of NA7Nef and SF2Nef, the structured cores accounted for the allelic variation.

### Is Nef oligomerization inhibited by the N-terminal myristate group?

To assess whether or not Nef oligomerization is regulated by membrane association, we determined the fractional dimerization of Nef from the membrane-bound and cytosolic fractions with the immunoprecipitation assay. Nef oligomers were demonstrated in both of these two compartments with the fraction of oligomeric Nef approximately two-fold higher in the membrane compartment. The immunoprecipitation assay should reflect the in vivo state of Nef except for very weakly associated Nef that would disassociate during cell lysis and immunoprecipitation. We have shown that the short HF tag does not generally interfere with Nef function though larger tags do in some instances [31]. Importantly, immunoprecipitation from cellular extracts would be expected to suppress non-specific interactions including aggregation of Nef protomers [44]. Thus, our observations are distinct from the previous observations of very weak self-association by purified Nef from bacteria (Kd > 0.5 mM) [10,45,46]. It is not clear that the weak association observed in vitro is reflective of functionally significant interactions inside cells.

The presence of Nef oligomers in the cytosol argues that Nef oligomerization is not strongly inhibited by the myristoylated N-terminal arm of Nef. If the binding of the N-terminal arm of Nef to the core blocked oligomerization, then the intramolecular nature of this interaction would overwhelm any weak tendency to self-association unless the N-terminal arm is dissociated from the core by stable binding to membrane [47-49]. These considerations argue strongly that a negative regulation of Nef oligomerzation by its N-terminal segment would result in cytosolic Nef being entirely monomeric. Our observation that relatively high levels of dimers are present in the cytosol demonstrates that the N-terminal domain does not significantly regulate dimerization and suggests that self-association of Nef is a constitutive property of the protein [10,14,18]. In the absence of data demonstrating sequestration of Nef oligomers to membranes, we conclude that Nef is unlikely to be sequentially regulated. Instead Nef appears to be in equilibrium between monomeric and oligomeric forms.

### Is oligomerization evolutionarily conserved?

We addressed the question of evolutionary conservation of Nef oligomerization by investigating whether or not SIV_MAC239_Nef oligomerized and whether SIV_MAC239_Nef and SF2Nef associate. Our positive findings for both cases demonstrate the existence of a conserved mechanism of dimerization. Although the location of the dimerization interface for this heterologous association is not known, it is certainly expected that it will be within a highly conserved region. This is in contrast to several other shared functions of HIV-1Nefs and SIVNefs with variant structural bases [2,28]. From this evolutionary perspective, conserved but mechanistically distinct activities like CD4 downregulation and MHCI downregulation would then represent evolutionarily malleable functions relative to oligomerization.

### What regions of Nef interact to form multimers?

The interface or interfaces responsible for Nef oligomerization have not yet been defined. Previously proposed dimerization interfaces within the Nef core are not sufficiently conserved between SIVNefs and HIV-1Nefs to be operative in these assays [10,11,18]. Nonetheless, our results do suggest the Nef core as the site of Nef dimerization interfaces. The Nef core exhibits the highest level of identity between SF2Nef and SIV_MAC239_Nef, especially the region largely encoded by nucleotides of the polypurine tract (amino acids, 88-99, in HIV-1Nef with 11 out of 12 identical). Also, our finding that dimer formation by the SF2Nef core is strong relative to the NA7Nef core further supports this conclusion (Figure 5B).

It does not seem likely that either the N-terminal arm of Nef or the internal flexible loop of Nef are involved in dimerization as they are weakly conserved between HIV-1Nef and SIVNef. The myristoylated N-terminal Nef synthetic peptide (amino acids 2-57) does not dimerize [45], and we have observed dimerization of SF2NefΔ160-175 despite removal of much of the internal loop (not shown). Therefore, Nef dimerization domains appear to be located in the highly conserved core of Nef [9]. These considerations validate the use of Nef structures from crystals, even though the Nefs are truncated for the N-terminal arm and flexible internal loop.

ClusPro analysis of HIV-1Nef homodimers and the HIV-1Nef and SIVNef heterodimers suggests a candidate interface for Nef dimerization. The critical residues of this proposed interface are L100, I101, R105, R106, I109, L112, W113, and H116. Four considerations support this interface. First, the residues are conserved, especially R106, I109, L112, and W113. Second, D123 is excluded from the model. Third, R105 and R106 interact within a region that is highly conserved between HIV-1Nef and SIVNef (HIV-1Nef residues 88-99). Fourth, ClusPro models for HIV-1Nef homodimers and HIV-1Nef/SIVNef heterodimers exhibit nearly identical binding (Figure 8). Extensive mutational and phenotypic analysis will be required to establish this model.

### How many oligomeric forms of Nef exist?

The existence of one or more dimerization domains distinct from the αB plus flanking residues does not exclude the presence of Nef dimers linked at this site. In fact, Poe and Smithgall have provided evidence that Nefs linked by this interface exists in intact cells [13]. However, our inability to demonstrate a role for the R105-D123 interface by formaldehyde cross-linking assays suggests that the dimer detected by Poe and Smithgall is a quantitatively minor form of total Nef dimers. In this regard, the biomolecular fluorescence complementation assays employed by Poe and Smithgard freezes in place the conformations of Nef that allow the molecular complementation of the N-terminal 85 amino acids of GFP to the C-terminal 153 amino acids of GFP. Once formed, the reconstituted GFP may block the dynamic equilibrium between Nef conformers and result in the accumulation of a minor form [13]. Another HIV-1Nef dimer that could be a minor form is the BM[PEG]_3 _cross-linked dimer. A second crystal interface could account for this interaction in the C-terminal region (Figure 9B, *Magenta *and *Lilac*).

### Interaction between SIVNefs and HIV-1Nefs with host cell proteins

There are minor Nef complexes that run above the SF2Nef dimer band in SDS/PAGE following formaldehyde cross-linking (See Figure 5). Whether these bands represent different forms of lysine cross-linked Nef with an altered SDS/PAGE mobility or Nef cross-linked to a different protein remains to be determined. However, the situation for SIV_MAC239_Nef appears to be different. Two prominent species of cross-linked SIV_MAC239_Nef are evident in Figure 5A, *right*. The lower of the two bands at approximately 82 kD could plausibly be a heterodimer of SIV_MAC239_-HFNef and a host cell protein. Alternatively, the different mobilities could result from variant lysine cross-links of SIV_MAC239_Nef dimers. We favor the latter interpretation for the following reasons. Of the various proteins shown to bind tightly to Nef only Rac1 (211 amino acids) is approximately the correct size [29,30]. Skowronski and co-workers report that Rac1 immunoprecipitates with NA7-HFNef and SIV_MAC239_Nef. As a result, either the tagged SF2-HFNef or the untagged SF2Nef in a cross-linked heterodimer with Rac-1 should migrate separately from SF2Nef dimer in SDS/PAGE. This is not the case (Figure 5A and 5C). Therefore, we conclude that the major Nef binding protein in intact cells is Nef.

The density of potential protein/protein interaction domains displayed in Figure 9 suggests that Nef is capable of initiating complex host cell associations in which two host cell proteins are brought together to achieve abnormal situations within the infected cell that are advantageous to the virus. Two ternary complexes have been recently reported between Nef, the cytoplasmic tail of MHCI, and AP-1, and Nef, the cytoplasmic tail of CD4, and AP-2 [40-42]. These interactions are proposed to downregulate cell surface expression or MHCI and CD4. Another interesting mechanistic explanation for explaning Nef functional complexity is the possibility of allosteric interactions [50]. Finally, Nef self-association may create new interfaces for protein association with each Nef contributing part of the interface. All of these mechanisms should be considered in efforts to resolve the complexities of Nef function made possible by the fastest genome evolution ever described [51].

## Conclusions

We have demonstrated significant levels of oligomerization of Nef in cells. This property of Nef is conserved between HIV-1Nef and SIVNef which strongly suggests functional significance. We found that the R105D mutation greatly reduced the fraction of dimeric Nef, but exhibited only partial defects in MHCI downregulation and PAK2 activation, and only a small effect on CD4 downregulation (Figure 7). For this reason, we favor the explanation that the functional role of Nef dimerization is a function not presently known.

Our results are contrary to a regulatory model in which Nef is induced by membrane binding to produce oligomers [14,15]. These models require cytosolic Nef to be monomeric with Nef dimers residing strictly on membranes, but we did not observe this to be the case. We further observed that the myristoylation-defective G2A mutation does not enhance oligomerization. For this reason it does not appear that Nef's association with membrane and Nef dimerization are mechanistically related. Since Nef exhibits different functions in multiple cellular compartments including the Golgi, endosomes, plasma membrane and cytosol, it logically follows that multiple conformers and oligomers of Nef would all have extensive distributions with monomers as well as oligomers being functionally active [1]. The existence of multiple oligomeric forms could greatly expand the activities exhibited by this small protein. Here we found that a major contributor to one or more Nef dimeric interfaces is the dibasic motif R105-R106. In the future it will be important to not only consider Nef as acting at specific cellular locations to elicit specific derangements in host cell physiology but also as acting in an expanded role throughout the cell.

## Methods

### Cell lines and culture conditions

293T cells were maintained in Dulbecco's modified Eagle's medium (DMEM; Cellgro, Herndon, VA) supplemented with 10% fetal bovine serum (FBS; Cellgro), 100 IU/ml of penicillin, 100 μg/ml streptomycin, 2 mM glutamine (Cellgro) in 10% CO_2 _at 37°C. CEM cells were cultured in RPMI 1640 medium supplemented with 5% FBS, 100 IU/ml penicillin, 100 μg/ml streptomycin, 2 mM L-glutamine, and 1 mM sodium pyruvate (Cellgro) and were maintained at 37 °C in a humidified incubator with 5% CO_2_.

### Expression vectors

pCG and pCGCG expression vectors encoding two wild type HIV-1Nefs, NA7Nef and SF2Nef, and HF-tagged Nefs, NA7-HFNef and SF2-HFNef, were described in Raney et al. [31]. pCG, pCGCG, and pCG239ahf (SIV_MAC239_Nef with the HF C-terminal tag) were provided by Jacek Skowronski [29,30]. The HF tag contains the HA epitope, YPYDVPDYA. pCG and pCGCG give equivalent expression of Nef. SF2Nef oligomerization mutants (R105D, D123R, R105D/D123R, R105A and R106A) and cysteine mutants (C55S, C142S, C206X), myristoylation-defective mutant G2A, and the Nef deletion mutant Δ160-175 were constructed using a site-directed mutagenesis kit (Stratagene) with oligonucleotides designed for each mutation. All mutated Nefs were confirmed by DNA sequencing. NA7/SF2 Nef chimeras were made by cloning the BlpI-BspEI fragment from SF2Nef (coding for amino acids 24-200) into pCGCGNA7Nef and vice versa. SF2Nef is four amino acids longer than the canonical 206 amino acids. To avoid confusion the position of amino acid residues are referenced to a canonical length of 206 amino acids [8].

### Co-immunoprecipitation and Western blot analysis

Expression vectors encoding NA7Nef, NA7-HFNef, SF2Nef, SF2-HFNef, SIV_MAC239_Nef, and SIV_MAC239_-HFNef were transfected into 293T cells in 6-well plates with Lipofectamine 2000 (Invitrogen, Carlsbad, CA). Cells were washed in cold phosphate buffered saline (PBS) then removed from the plate in 1 ml of 25 mM Tris-HCl, pH = 7.5, 150 mM NaCl, and 5 mM EDTA (Buffer A). Cell suspensions were disrupted by sonication (ten, 10 second bursts at power level 3.5) at 4°C. Nuclei were removed by centrifugation at 800 Xg for 10 minutes. The low speed supernatant fraction was re-spun at 27,000 rpm for 25 minutes in a MLA 130 fixed angle rotor (Beckman). The high speed supernatant fraction was separated from the pellet and adjusted to 1 ml to contain 50 mM Tris, pH = 8.0, 100 mM NaCl, 25 mM NaF, 25 mM benzamidine, 20 mM β-glycerophosphate, 2 mM Na_3_VO_2_, 3 mM EDTA, 10% glycerol, plus Roche protease inhibitors (Buffer B). The buffer-adjusted high speed supernatant sample was used to determine total soluble Nef expression (100 μl) and for immunprecipitation studies (900 μl). The pellet from the high speed centrifugation was rinsed with 0.5 ml of Buffer A. The membrane pellet was then re-suspended in 1 ml of Buffer A by Dounce homogenization and spun at 27,000 rpm. The pellet was saved and solubilized in 1 ml of Buffer B plus 0.5% IGEPAL CA-630 with a Dounce homogenizer and respun at 27,000 rpm for 10 minutes. The supernatant fraction of the detergent solubilized membrane pellet was used for determining total membrane-bound Nef expression (100 μl) and for immunoprecipitation studies (900 μl). With this procedure there was no detectable contamination of soluble protein in the membrane fraction or membrane protein in the soluble fraction (Additional file 2; Figure S2, *Left *and *Middle*). The distribution of membrane-bound and soluble SF2Nef and membrane-bound and soluble SF2NefG2A were nearly the same as previously reported values (Additional file 2; Figure S2, *Right*). Nef proteins were detected by Western blot analysis. Three monoclonal antibodies were used for detection: EH1 from James Hoxie [52], anti-SIV Nef 17.2 from the AIDS Repository, anti-HA (Covance). Two polyclonal antibodies were used for immunoprecipitations: sheep anti-SF2Nef [43] and sheep anti-SIV_MAC239_Nef antibody (Rogers, RP, Foster, JL and Garcia JV, unpublished.) Bands were quantitated by scanning the autoradiograms and determining density using ImageJ (Rasband, W.S., ImageJ, U. S. National Institutes of Health, Bethesda, Maryland, USA, http://rsb.info.nih.gov/ij/, 1997-2009). Density values are reported as the ImageJ value divided by 1000 to simplify the presentation.

### Nef cross-linking by formaldehyde or 1,11-bis(maleimido)triethylene glycol (BM[PEG]_3_)

For formaldehyde-mediated Nef cross-linking, expression vectors encoding wild type or Nef mutants were transfected into 293T cells. Prior to harvest intact cells were incubated with or without formaldehyde for 10 min at room temperature and washed with PBS. Then, whole cell lysates were prepared in Buffer B plus 0.5% IGEPAL CA-630, centrifuged at 13,000 Xg for 30 minutes and the supernatant fraction collected. Cross-linking of Nef by BM[PEG]_3 _(Pierce) was performed as follows: Transfected 293T cells were lysed in Buffer B plus 0.5% IGEPAL-630 and the lysate centrifuged. Clarified lysates were incubated with or without BM[PEG]_3 _dissolved in DMSO for 1 h at room temperature. The analysis of Nef cross-linking by formaldehyde (at lysine residues) or BM[PEG]_3 _(at cysteine residues) was performed by SDS-PAGE Western blotting.

### Retrovirus vector preparation, transduction and flow cytometry analysis

293T cells were transfected with the amphotropic packaging vector pEQPAM and pLXSN (vector control) or appropriate pLNefSN vectors with Lipofectamine 2000 (Invtrogen). Medium was harvested approximately 48 hours post-transfection and was filtered through a 0.45 μm filter.

A 24-well plate was coated with 40 μg/well of retronectin (Takara Biomedicals, Kyoto, Japan). After 2 h at room temperature, the retronectin was removed, 0.5 ml 2% bovine serum albumin in PBS was added for 30 min at room temperature, and the wells were washed once with PBS (0.5 ml). Filtrate (0.5 ml) containing amphotrophic vector was then added and left on the plate for 45 min at 37°C, and this procedure was repeated. CEM cells (300,000) were transduced by the retronectin-bound retroviral vector in 0.5 ml of complete RPMI overnight at 37°C. An additional 0.5 ml of vector was added the next day. On the following day, the cells were re-suspended in a 12-well plate containing 1.5 mg/ml G418 (Gibco) in a final volume of 2 ml. After 48 hours, the cells were re-plated in a 6-well plate and 1 ml of medium plus G418. Transduced cells then were expanded into a T75 flask without G418 for subsequent analysis.

The transduced CEM cells were labeled for analysis of cell surface CD4 and MHC-1 levels. Cells (500,000) were first incubated with a mouse monoclonal antibody recognizing haplotypes A1, A11, and A26 of MHC class 1 (One Lambda, Canoga Park, CA) for 20 min on ice in the dark, and then the cells were washed twice in 2 ml of ice-cold PBS containing 5% FBS. Cells were then incubated with fluorescein isothiocyanate (FITC)-labeled goat anti-mouse IgG for 20 min on ice. Cells were washed as indicated above and incubated for 20 min on ice with 2 μg of mouse IgG, and washed again. Cells were then incubated with phycoerythrin (PE)-conjugated IgG monoclonal antibody to human CD4 (Exalpha, Maynard, MA) for 20 min on ice. Stained cells were washed and analyzed on a Becton Dickinson FACSCalibur instrument equipped with Cellquest-Pro software. All fluorescence data were collected in log mode. CEM cells transduced with LXSN served as the positive control for CD4 and MHCI cell surface expression. For negative controls, mouse isotype antibody replaced anti-MHC class I, and PE-conjugated mouse IgG (Exalpha) replaced PE-conjugated anti-CD4.

## Competing interests

The authors declare that they have no competing interests.

## Authors' contributions

YTK, AR, LSK, and SJD performed experiments. SJD quantitated autoradiographs by ImageJ, performed analysis with ClusPro and prepared figures. BSRT directed ClusPro analysis. JVG and JLF wrote the paper. All authors read and approved the final manuscript.

## Supplementary Material

Additional file 1 **Figure S1- Cross-reactivity of NA7-HFNef and SF2-HFNef in Western blot analysis**. **(A)**, NA7-HFNef and SF2-HFNef were expressed in 293T cells by transient transfection. Whole cell extracts were prepared and subjected to SDS/PAGE. Three separate Western blots were prepared and Nef detected with three separate antibodies. Lanes 1-3, Vector control, NA7-HFNef, and SF2-HFNef, respectively. *Left*, Blot was probed with monoclonal antibody EH1 (α-Nef_EH1_). This antibody binds to the thirteen C-terminal amino acids of SF2Nef (*52*). Since the last 18 amino acids of SF2Nef and NA7Nef are identical it is expected that the interaction between these two proteins and EH1 would be identical. *Middle*, blot probed with sheep anti-Nef (sheep α-Nef) *Right*, Blot probed with monoclonal anti-HA (α-HA). **(B)**, Quantitation of the Nef bands was performed with ImageJ and the ratio of NA7-HFNef density to SF2-HFNef density was determined for each antibody. Note that α-HA and α-Nef_EH1 _by virtue of recognizing identical epitopes allow calculation of the relative levels of expression of the two Nefs while the ratio of NA7-HFNef to SF2-HFNef densities will represent differential protein expression and any difference in the ability of the sheep anti-Nef polyclonal antibody to detect the two Nefs. The ratio of the densities for NA7-HFNef/SF2-HFNef was determined to be 0.69 for sheep anti-Nef. The ratios for monoclonal EH1 and anti-HA were 0.86 and 0.75, respectively giving an average of 0.805 (NA7Nef/SF2Nef). Dividing the NA7Nef/SF2Nef ratio for sheep anti-Nef (0.69) by the estimated difference in expression between NA7-HFNef and SF2-HFNef (0.805) in this transfection gives the fractional binding of sheep anti-Nef to NA7Nef relative to SF2Nef which is 0.86. In other words a 14% reduction in binding is observed for sheep anti-Nef for NA7Nef relative to SF2Nef. The dominant epitopes for this polyclonal antibody reside between SF2Nef amino acids 17-110 (*54*). NA7Nef and SF2Nef have only 9 differences within this region (not shown). Click here for file

Additional file 2 **Figure S2- Distribution of SF2Nef and SF2G2A in membrane and cytosolic compartments**. 293T cells were transfected with pCGSF2Nef and pCGSF2NefG2A. Cells that were not transfected served as the negative control. The three cell samples were processed for membrane and soluble fractions as described in **Methods**. The final fractions were adjusted to contain total membrane protein (330 ± 60 μg, average of three samples) and soluble protein (860 ± 130 μg, average of three samples) each in a total volume of 1 ml. Equal aliquots of the two fractions from each sample were analyzed by SDS/PAGE. *Left*- Lanes 1-3, Soluble fractions; Lanes 4-6, Membrane fractions. Lanes 1 and 4, pCGSF2-HFNef; Lanes 2 and 5, pCGSF2-HFNefG2A; Lanes 3 and 6, not transfected. Western blot analysis performed with antibody to the strictly membrane associated cadherins (α-Pan Cadherin). *Middle*- same as *Left *except Western blot analysis performed with antibody to the strictly soluble GAPDH (α-GAPDH). *Right*- same as *Left *except Western blot with antibody to Nef (α-Nef). Quantification by ImageJ determined that membrane-bound SF2-HFNef was 34% and soluble SF2-HFNef was 66% of total Nef. Membrane-bound and soluble SF2-HFNefG2A was 8% and 92%, respectively. These values are consistent with previously reported values for NL4-3Nef and NL4-3NefG2A in HeLa cells (*16*). Click here for file

Additional file 3 **Figure S3- Oxidation of Nef in extra-cellular extracts**. To confirm previously published results we subjected solubilized whole cell extracts containing SF2Nef, NA7Nef, or SIV_MAC239_Nef to oxidizing conditions. Extracts were prepared as described in **Methods **for immunoprecipitation without β-mercaptoethanol. Following sonication the samples were not fractionated but instead detergent containing buffer added to give solubilized whole cell extracts followed by centrifugation. Supernatant samples were prepared for SDS/PAGE by boiling in SDS sample buffer with (+) and without (-) β-mercaptoethanol. Western blots were then developed with either anti-HIV-1Nef (α-HIVNef) or anti-SIVNef (α-SIVNef). Lanes 1, 4, 7, and 10 are SF2Nef; Lanes 2, 5, 8, and 11 are NA7Nef; Lanes 3, 6, 9, and 12 are SIV_MAC239_Nef. Lanes 1-3 and 7-9 are samples boiled in SDS sample buffer with β-mercaptoethanol; Lanes 4-6 and 10-12 are samples boiled in SDS sample buffer without β-mercaptoethanol. Click here for file
