# Supplementary figures and images for: Self-association of the Lentivirus protein, Nef
Source: Retrovirology. 2010 Sep 23;7:77. doi: 10.1186/1742-4690-7-77 (PMC2955668; doi:10.1186/1742-4690-7-77)

Supplementary Figure 1.

A.

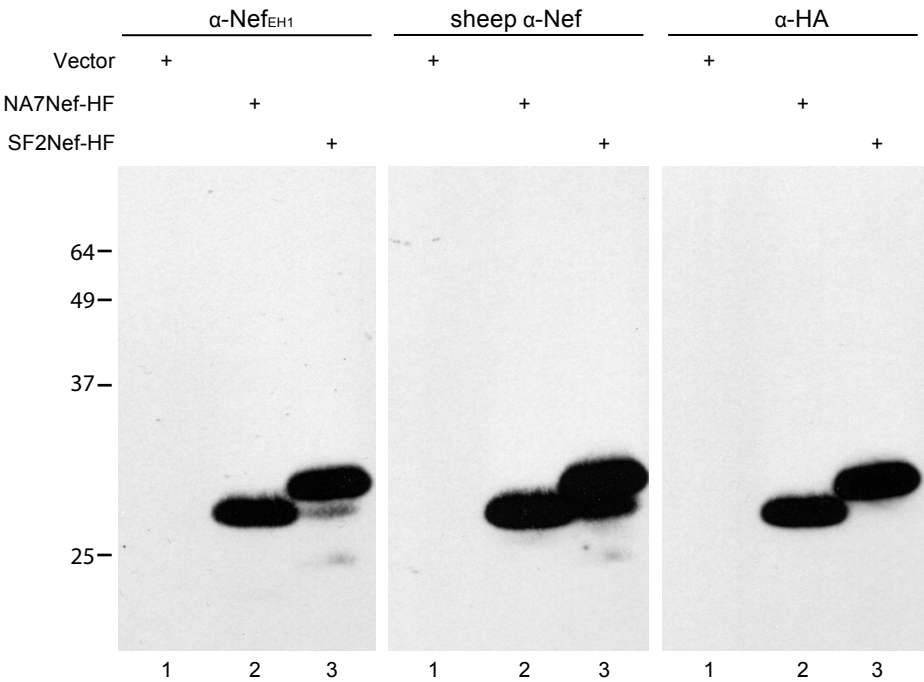

B.

|           | $\alpha$ -Nef <sub>EH1</sub> | sheep $\alpha$ -Nef | $\alpha$ -HA |
|-----------|------------------------------|---------------------|--------------|
| NA7Nef-HF | 2386                         | 3147                | 2576         |
| SF2Nef-HF | 3178                         | 4579                | 3000         |
| Ratio     | 0.75                         | 0.69                | 0.86         |

Supplement: Additional file 1 — Figure S1- Cross-reactivity of NA7-HFNef and SF2-HFNef in Western blot analysis. (A), NA7-HFNef and SF2-HFNef were expressed in 293T cells by transient transfection. Whole cell extracts were prepared and subjected to SDS/PAGE. Three separate Western blots were prepared and Nef detected with three separate antibodies. Lanes 1-3, Vector control, NA7-HFNef, and SF2-HFNef, respectively. Left, Blot was probed with monoclonal antibody EH1 (α-NefEH1). This antibody binds to the thirteen C-terminal amino acids of SF2Nef (52). Since the last 18 amino acids of SF2Nef and NA7Nef are identical it is expected that the interaction between these two proteins and EH1 would be identical. Middle, blot probed with sheep anti-Nef (sheep α-Nef) Right, Blot probed with monoclonal anti-HA (α-HA). (B), Quantitation of the Nef bands was performed with ImageJ and the ratio of NA7-HFNef density to SF2-HFNef density was determined for each antibody. Note that α-HA and α-NefEH1 by virtue of recognizing identical epitopes allow calculation of the relative levels of expression of the two Nefs while the ratio of NA7-HFNef to SF2-HFNef densities will represent differential protein expression and any difference in the ability of the sheep anti-Nef polyclonal antibody to detect the two Nefs. The ratio of the densities for NA7-HFNef/SF2-HFNef was determined to be 0.69 for sheep anti-Nef. The ratios for monoclonal EH1 and anti-HA were 0.86 and 0.75, respectively giving an average of 0.805 (NA7Nef/SF2Nef). Dividing the NA7Nef/SF2Nef ratio for sheep anti-Nef (0.69) by the estimated difference in expression between NA7-HFNef and SF2-HFNef (0.805) in this transfection gives the fractional binding of sheep anti-Nef to NA7Nef relative to SF2Nef which is 0.86. In other words a 14% reduction in binding is observed for sheep anti-Nef for NA7Nef relative to SF2Nef. The dominant epitopes for this polyclonal antibody reside between SF2Nef amino acids 17-110 (54). NA7Nef and SF2Nef have only 9 differences within th [file 1742-4690-7-77-S1.PDF]

Supplementary Figure 2.

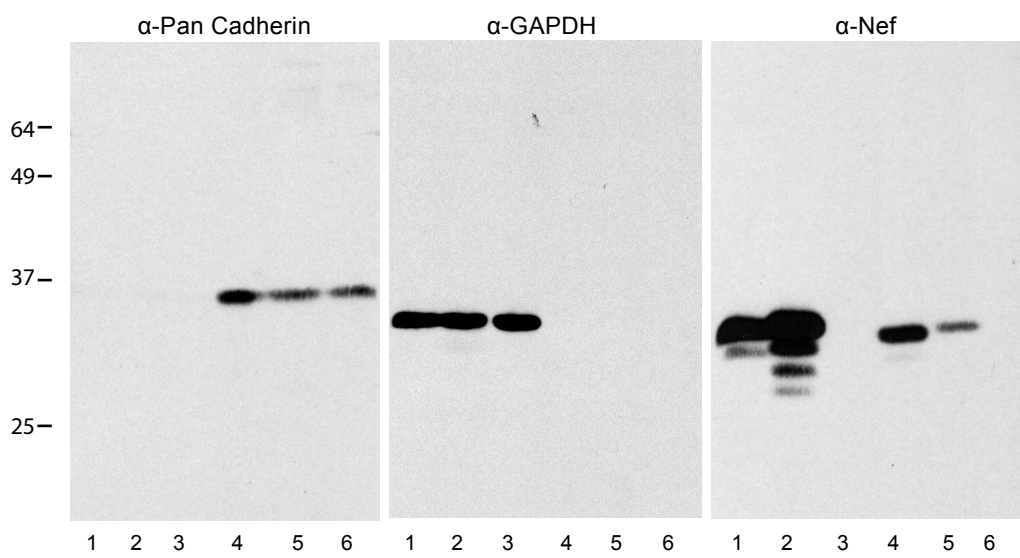

Supplement: Additional file 2 — Figure S2- Distribution of SF2Nef and SF2G2A in membrane and cytosolic compartments. 293T cells were transfected with pCGSF2Nef and pCGSF2NefG2A. Cells that were not transfected served as the negative control. The three cell samples were processed for membrane and soluble fractions as described in Methods. The final fractions were adjusted to contain total membrane protein (330 ± 60 μg, average of three samples) and soluble protein (860 ± 130 μg, average of three samples) each in a total volume of 1 ml. Equal aliquots of the two fractions from each sample were analyzed by SDS/PAGE. Left- Lanes 1-3, Soluble fractions; Lanes 4-6, Membrane fractions. Lanes 1 and 4, pCGSF2-HFNef; Lanes 2 and 5, pCGSF2-HFNefG2A; Lanes 3 and 6, not transfected. Western blot analysis performed with antibody to the strictly membrane associated cadherins (α-Pan Cadherin). Middle- same as Left except Western blot analysis performed with antibody to the strictly soluble GAPDH (α-GAPDH). Right- same as Left except Western blot with antibody to Nef (α-Nef). Quantification by ImageJ determined that membrane-bound SF2-HFNef was 34% and soluble SF2-HFNef was 66% of total Nef. Membrane-bound and soluble SF2-HFNefG2A was 8% and 92%, respectively. These values are consistent with previously reported values for NL4-3Nef and NL4-3NefG2A in HeLa cells (16). [file 1742-4690-7-77-S2.PDF]

Supplementary Figure 3

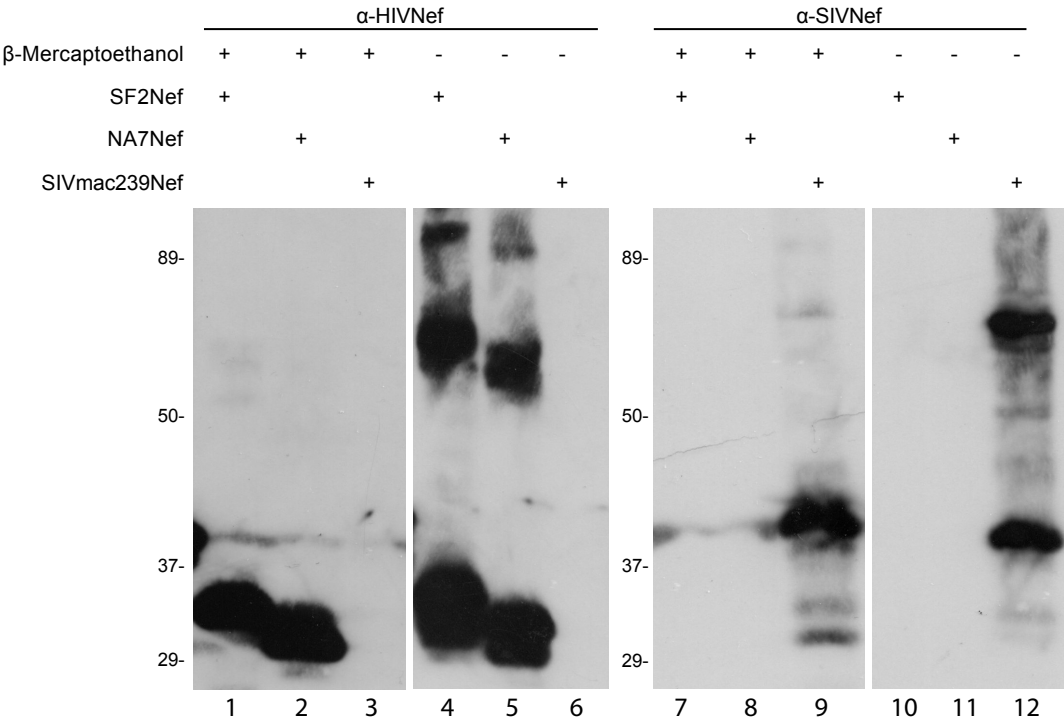

Supplement: Additional file 3 — Figure S3- Oxidation of Nef in extra-cellular extracts. To confirm previously published results we subjected solubilized whole cell extracts containing SF2Nef, NA7Nef, or SIVMAC239Nef to oxidizing conditions. Extracts were prepared as described in Methods for immunoprecipitation without β-mercaptoethanol. Following sonication the samples were not fractionated but instead detergent containing buffer added to give solubilized whole cell extracts followed by centrifugation. Supernatant samples were prepared for SDS/PAGE by boiling in SDS sample buffer with (+) and without (-) β-mercaptoethanol. Western blots were then developed with either anti-HIV-1Nef (α-HIVNef) or anti-SIVNef (α-SIVNef). Lanes 1, 4, 7, and 10 are SF2Nef; Lanes 2, 5, 8, and 11 are NA7Nef; Lanes 3, 6, 9, and 12 are SIVMAC239Nef. Lanes 1-3 and 7-9 are samples boiled in SDS sample buffer with β-mercaptoethanol; Lanes 4-6 and 10-12 are samples boiled in SDS sample buffer without β-mercaptoethanol. [file 1742-4690-7-77-S3.PDF]
